# Using a Partially Evaporating Cycle to Improve the Volume Ratio Problem of the Trilateral Flash Cycle for Low-Grade Heat Recovery

**DOI:** 10.3390/e23050515

**Published:** 2021-04-23

**Authors:** Kai-Yuan Lai, Yu-Tang Lee, Ta-Hua Lai, Yao-Hsien Liu

**Affiliations:** 1 Department of Mechanical Engineering, National Chiao Tung University, 1001 Ta-Hsueh Road, Hsinchu 30010, Taiwan; itria20076@itri.org.tw (K.-Y.L.); leeadam0710.me07g@nctu.edu.tw (Y.-T.L.); 2 Green Energy and Environment Research Laboratories, Industrial Research Institute (ITRI), Sec. 4, Zhongxing Road, Hsinchu 31057, Taiwan; DerekTHL@itri.org.tw

**Keywords:** industrial waste heat, partially evaporating cycle, trilateral flash cycle, volume ratio, low-grade heat recovery

## Abstract

This study examined the trilateral flash cycle characteristics (TFC) and partially evaporating cycle (PEC) using a low-grade heat source at 80 °C. The evaporation temperature and mass flow rate of the working fluids and the expander inlet’s quality were optimized through pinch point observation. This can help advance methods in determining the best design points and their operating conditions. The results indicated the partially evaporating cycle could solve the high-volume ratio problem without sacrificing the net power and thermal efficiency performance. When the system operation’s saturation temperature decreased by 10 °C, the net power, thermal efficiency, and volume ratio of the trilateral flash cycle system decreased by approximately 20%. Conversely, with the same operational conditions, the net power and thermal efficiency of the partially evaporating cycle system decreased by only approximately 3%; however, the volume ratio decreased by more than 50%. When the system operating temperature was under 63 °C, each fluid’s volume ratio could decrease to approximately 5. The problem of high excessive expansion would be solved from the features of the partially evaporating cycle, and it will keep the ideal power generation efficiency and improve expander manufacturing.

## 1. Introduction

Low-grade waste heat generally pertains to continuous emission and requires no fuel cost. Moreover, low-grade waste heat under 100 °C represents 63% of the global waste heat [1]. Substantial recovery of waste and residual heat for power generation can greatly facilitate energy conservation and reduce carbon emissions. For governments, this can largely contribute to the power load of electrical grids. The organic Rankine cycle (ORC) proposed by Tabor and Bronicki [2,3] uses an organic fluid as a working fluid that absorbs waste heat to generate power. The product’s strengths include its maturity in technique, high reliability, and low cost; therefore, it is a standard solution with higher transformation efficiency for power generation with medium- and low-grade residual heat [4]. This technique is widely used for waste heat recovery in plants [5] and geothermal power generation [6,7]. ORC is one of the most widely used technologies for waste heat recovery in Europe, the United States, and Central America [8]. However, during heat extraction in the ORC system, the working fluid undergoes phase variation, making no good thermal match between the working fluid and the heat source, which disables the effective and full utilization of the heat source [9].

**Figure 1:**
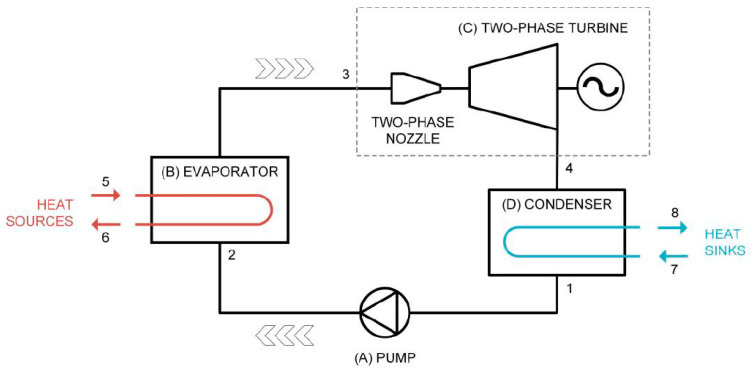
Schematic diagram of the TFC and PEC cycle system components.

To facilitate heat transfers between the heat source and the working fluid, Smith et al. [10] proposed a thermodynamic cycle—the trilateral flash cycle (TFC)—in 1993. Figure 1 presents its schematic diagram. The entire system is divided into four parts: (A) The pressure-increasing component in which a pump is used to increase the pressure of low-pressure organic working fluid and deliver the fluid to the component for heat extraction; (B) the heat extraction component in which an evaporator is used to extract heat energy at the end of the heat source to heat the organic working fluid in the loop system with the system pressure controlled to retain the working fluid in a liquid state during the heat exchange; (C) the heat work conversion component in which a two-phase turbine is used to convert heat energy and pressure energy from the organic working fluid into the shaft work, with the shaft work yielding power through a generator and the working fluid leaving the turbine in a low-pressure two-phase turbine from following the work done on the heat work conversion component; and (D) the component of heat rejection in which a condenser is used to remove the gaseous heat energy of the organic working fluid that is condensed into liquid to accomplish the thermodynamic cycle of the TFC system, which does not enter the two-phase region during heat absorption.

**Figure 2:**
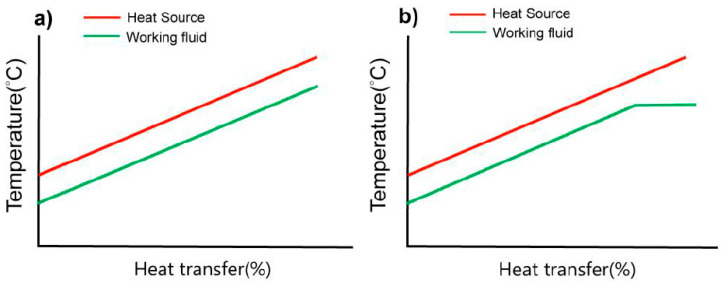
(**a**) Temperature—heat transfer diagram for the TFC. (**b**) Temperature—heat transfer diagram for the PEC.

Figure 2a illustrates the temperature variation of the heat exchanger and indicates that both sides’ temperature variations are nearly parallel. The heat extraction method can resolve the pinch point found in the ORC. Although this method’s thermal efficiency is slightly lower than that of the ORC [4], it can absorb heat more effectively from a heat source. In particular, with a low-grade heat source, the heat generation rate should be prioritized over thermal efficiency [11]. From the literature review, the heat energy recovered by TFC is much more than ORC under most circumstances [12]. Meanwhile, the TFC exhibits a more favorable power generation capacity under low-temperature heat source conditions (<80 °C) [13].

The expansion process of the TFC cycle occurs when a high-pressure saturated liquid expands into a low-pressure two-phase fluid. The saturated liquid’s high density in a pure liquid state results in an excessively high volume ratio. Excessive volume change reduces the expander’s efficiency and leads to manufacturing challenges. Table 1 lists the previous researches on the TFC’s volume ratio. Smith [10] indicates that the water volume ratio is too high in heat source temperature of 100 °C. Furthermore, the volume ratio of organic fluid is 30 times smaller than using water as the working fluid. Trædal [14] applies different organic fluids to analyze the performance in the same heat source and heat sink condition. The minimum volume ratio of the same fluid used in this study’s subsequent chapters is 11 using the working fluid of R134a. The volume ratios in Fischer’s [15] research are also high.

The saturation temperature of the cycle must be lowered to reduce the volume ratio [9]. Research by Bianchi et al. [16] revealed that when the quality of the inlet of the expander remains the same, the expander inlet temperature must be lowered to approximately 100 °C for the volume ratio of the working fluid R1233zd to decrease from 80 to less than 10. This indicated that lowering the system’s saturation temperature cannot effectively resolve the working fluid’s excessive volume ratio.

In recent years, scholars have proposed an improved cycle—the partially evaporating cycle (PEC) [17]—to resolve the excessively high volume ratio. The system components it requires are similar to those of the TFC, with the main difference being that an evaporator replaces a heat exchanger as a heat extraction component. The heat exchange is conducted using a working fluid with a slightly lower saturation pressure. Figure 2b presents the temperature–heat transfer diagram. During the heat exchange, part of the fluid is evaporated and leaves the evaporator in a two-phase manner. This method of partially evaporating the fluid can significantly reduce the volume ratio of working fluid during expansion. When only part of the fluid evaporates during the heat exchange (low quality), the temperature variations of the first sections of the curves of the two sides of the heat exchanger remain nearly parallel. This indicates that the PEC has a high heat extraction rate, which the TFC also possesses. Research by Lecompte [17] used a 140 °C heat source and R245fa as the working fluid for analysis, and the results indicated that when the quality of the expander inlet was less than 0.3, the amount of heat extracted was equal to that of the TFC state (x_3 = 0). However, when the quality increased to 0.45, the limit of the pinch point temperature difference (PPTD) caused the amount of heat extracted to decrease. Afterward, the system gradually transformed into the ORC as the quality increased. The phenomenon reflects the importance of observing the PPTD in the PEC system.

To explore the effect of the PPTD on the PEC system during heat extraction, low-grade waste heat was presumed in this study. Based on previous studies, the smallest temperature difference permitted by the heat source end was set as 5 °C [18,19]. By observing the pinch point, the evaporation temperature and mass flow rate of the working fluid and the quality of the expander inlet were adjusted to determine the most suitable working fluid and operational conditions for the TFC and PEC systems. Consequently, the two systems’ performances regarding the amount of power generated, thermal efficiency, and the volume ratio were compared.

## 2. Theoretical Modeling

**Figure 3:**
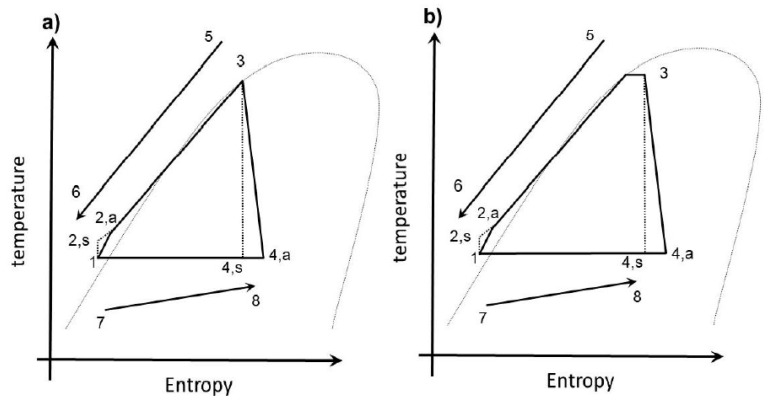
(**a**) T-s diagram for the TFC and (**b**) for the PEC.

In this study, the mathematical models of the TFC and PEC were established from the laws of thermodynamics and the thermal properties database REFPROP 9.0 [20]. Figure 3a displays the T-s diagram of a typical TFC. When the cycle began, the working fluid was a low-pressure saturated liquid (point 1) compressed by the pump and entered a high-pressure liquid state (point 2) before entering the heat exchanger and undergoing heat exchange with the heat source. After this was accomplished, the working fluid left the heat exchanger (point 3) in a high-pressure saturated state, entered the expander, and left in a low-pressure two-phase form (point 4). Finally, this was cooled down into its original state, thus accomplishing a cycle. Figure 3b is the T-s diagram for the thermodynamic part of the PEC. The most critical difference between the two was that the working fluid in the PEC had two phases—gaseous and liquid—at the heat exchanger outlet (point 3), whereas that of TFC was a saturated liquid because the working pressure of the PEC during heat exchange was slightly lower than that of the TFC, which led to the partial evaporation of the fluid during the heat exchange.

**Figure 4:**
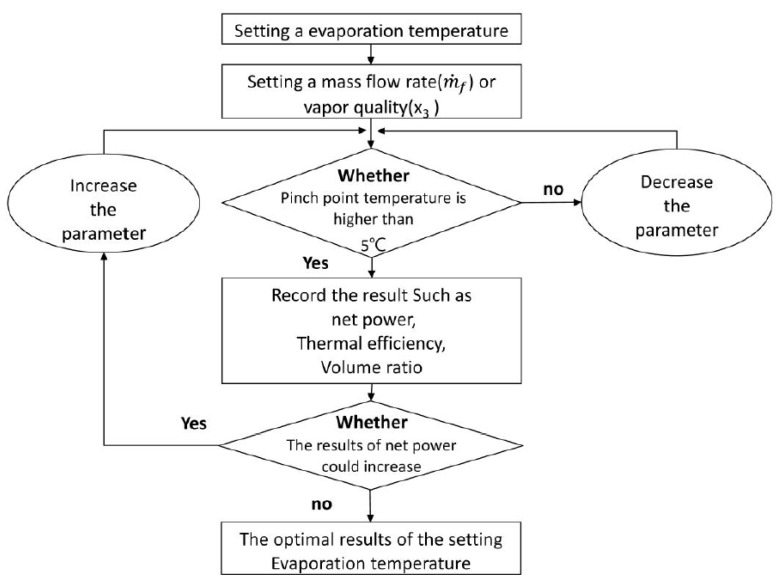
Flowchart of the simulation procedure.

In the beginning of the simulation, the temperature of the pump inlet was first set to 37 °C (point 1), and that of the cooling source was set to 30 °C (point 7), followed by setting the saturation pressure ($P_{2}$) of the heat exchanger and its corresponding variables by observing the variation of the PPTD in the model. The primary variable of the TFC is the mass flow rate (${\dot{m}}_{wf}$), whereas the PEC has an additional variable: heat exchanger outlet quality ($x_{3}$). The system was optimized with a fixed mass flow rate and a variable outlet vapor quality during the simulation. Figure 4 presents a schematic flowchart of this optimization. After obtaining the thermal properties of each point, pump power consumption can be obtained using the isentropic efficiency of the pump and the mass flow rate of the working fluid:

(1) $${\dot{W}}_{pup}=\frac{{\dot{m}}_{f}\left(h_{2,s}-h_{1}\right)}{\eta_{pup}}={\dot{m}}_{wf}\left(h_{2,a}-h_{1}\right)$$

This study presumed that no heat energy loss or pressure drop occurred in the heat exchanger section. For the TFC, the working fluid during the heat exchange remains a saturated liquid, and the location of the heat exchanger outlet can be obtained by directly setting the saturation pressure (point 3). The working fluid at the heat exchanger outlet of the PEC (point 3) has two phases—gaseous and liquid. Therefore, in addition to setting the saturation pressure, the quality of the heat exchanger outlet must be calculated. The amount of heat exchanged during the entire heating process can be obtained by the enthalpy change:

(2) $${\dot{Q}}_{H}={\dot{m}}_{wf}\left(h_{3}-h_{2,a}\right)={\dot{m}}_{H}\left(h_{6}-h_{5}\right)$$

When the working fluid leaves the heat exchanger and enters the expander for two-phase expansion, the amount of power generated (gross power) from the generator can be obtained from the enthalpy change and isentropic efficiency of the nozzle and rotors:

(3) $${\dot{W}}_{t}={\dot{m}}_{wf}\left(h_{3}-h_{4,s}\right)\eta_{nozzle}={\dot{m}}_{f}\left(h_{3}-h_{4,a}\right)$$

(4) $${\dot{W}}_{gross}={\dot{m}}_{t}\cdot \eta_{rotor}$$

where

(5) $$\eta_{nozzle}=0.865+0.00175\cdot d_{v}$$

(6) $$\eta_{rotor}=0.575+0.325\cdot x_{4}$$

$d_{v}$ represents the density of the saturated vapor under low-condensation pressure, and x represents the quality of the expander outlet. The efficiency formula of the two-phase nozzle is based on research by Welch et al. [21,22] and considers the major influencing properties of working fluids, such as vapor density and vapor mass.

With the same condenser and heat exchange components, as lack of heat energy loss and pressure decrease is assumed as:

(7) $${\dot{Q}}_{L}={\dot{m}}_{wf}\left(h_{4,a}-h_{1}\right)={\dot{m}}_{L}\left(h_{8}-h_{7}\right)$$

The thermal efficiency of the system can finally be calculated from the obtained net power generation and heat transfer:

(8) $${\dot{W}}_{net}={\dot{W}}_{gross}-{\dot{W}}_{pup}$$

(9) $$\eta_{th}=\frac{{\dot{W}}_{net}}{{\dot{Q}}_{H}}$$

The volume ratio of the two-phase expander can be obtained using the known properties of States 3 and 4.

(10) $$\gamma =\frac{d_{3}}{d_{4}}$$

Based on the heat transfer requirements and practical engineering considerations, the temperature difference between the heat source and working fluid is required for heat exchange. Due to cycle differences, the pinch point of the TFC is between the heat source outlet temperature and the heat exchanger inlet temperature at the side of the working fluid, whereas that of the PEC is between the heat source temperature and the evaporation temperature of the working fluid. To optimize the model, the pinch is considered a limiting condition during the simulation to calculate the optimal mass flow rate and best quality of each working fluid at different saturation temperatures.

**Figure 5:**
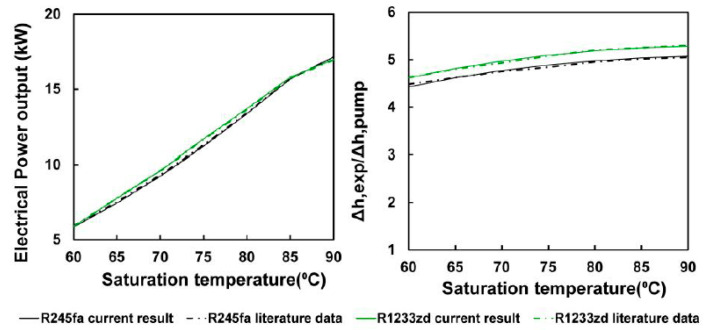
Comparison between the thermal analysis of this study with that of the literature [23].

To verify the constructed model, the same setting with that of research by McGinty et al. [23] was first conducted for the thermal model (Table 2), followed by a comparison of the thermal analysis results. Figure 5 displays the simulation results. The comparison chart indicated that the analysis results of the thermal model used in this study were similar to those of the literature.

## 3. Setting

Choosing a working fluid is critical to designing a waste heat recovery system. Its applicability depends on the evaporation temperature and condensation temperature of the system, which is decided by the temperature conditions of the cooling source and the heat source constructed by the system. Residual heat left from plant production was presumed to be the heat source in this study. Thus, 1 kg/s of 80 °C water was the heat source, and the cooling source was cold water at 30 °C. Organic working fluids such as R245fa, R134a, and R236fa [24], which are often used in heat recovery machine sets, were chosen for analysis in this study. In addition, this study analyzed the new refrigerant R1233zd aiming to replace R123, because of environmental requirements, and isopentane, a fluid that yields great power that has been generated in several studies on the TFC [25]. Table 3 lists the study parameters. The isentropic efficiency of the pump was set to 0.7 [13]. According to the research by Trædal [14], the isentropic efficiency of the nozzle and rotors were calculated through the above equations with the density and quality of the vapor as the variables, and no heat and pressure losses were presumed from the heat exchanger and condenser. The PPTDs between the heat source and evaporator and that between the heat source and condenser were 5 °C and 1 °C, respectively.

**Figure 6:**
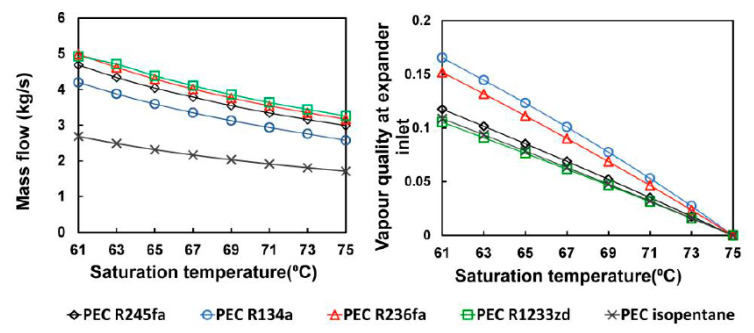
Suitable parameters for different saturation temperatures with different working fluids.

Figure 6 shows the simulation results, which indicated that the mass flow rate of the working fluid was negatively correlated with the saturation temperature because the amount of heat exchanged through the system increased as the evaporation temperature rose, which lowered the hot water outlet temperature and diminished the temperature difference with the working fluid. By then, the mass flow rate of the working fluid had to be reduced to preserve the limiting conditions of the simulation. In this study, the optimal mass flow rate of each working fluid at different saturation temperatures in the TFC was first calculated, and the mass flow rate that matched the maximum net power generation was considered for the setting parameter of the PEC. Similarly, the expander inlet quality at different saturation temperatures could then be calculated with the pinch point as the limiting condition.

**Figure 7:**
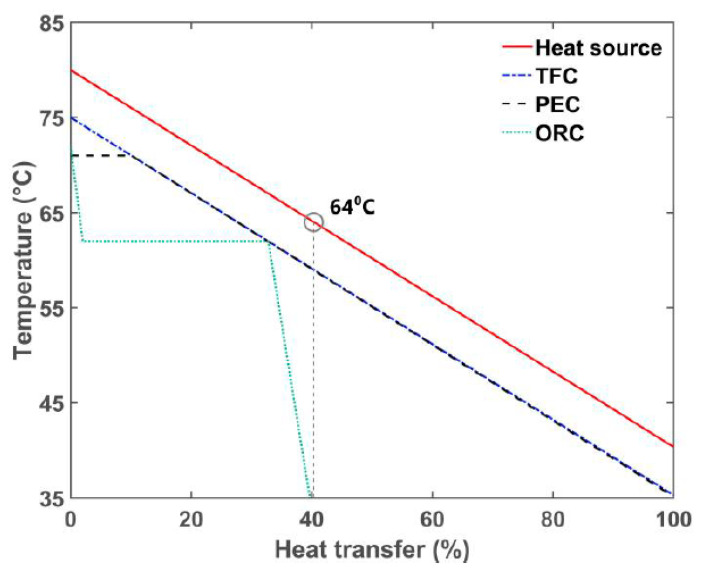
Comparison of heat transfer for the TFC, PEC, and ORC.

To understand the properties of the heat exchange processes of the TFC, PEC, and ORC, R245fa was taken as the working fluid in this study following the optimization of the simulation parameters, and the three types of heat exchange processes were compared. Figure 7 illustrates the simulation results, which reveals that the TFC hot water side temperature dropped to approximately 40 °C after the heat exchange was terminated. This indicated that this cycle could effectively obtain energy from the hot water side. For the ORC, the limit of the pinch point disabled the effective absorption of heat energy from the hot water side. When the heat exchange was terminated, the outlet temperature of the hot water side still reached 64 °C, and its utilization rate of usable energy was only 40% of that of the TFC. As for the PEC, partial evaporation sustained its high utilization rate of usable energy during the heat exchange, which is a property shared by the TFC.

## 4. Results and Discussion

**Figure 8:**
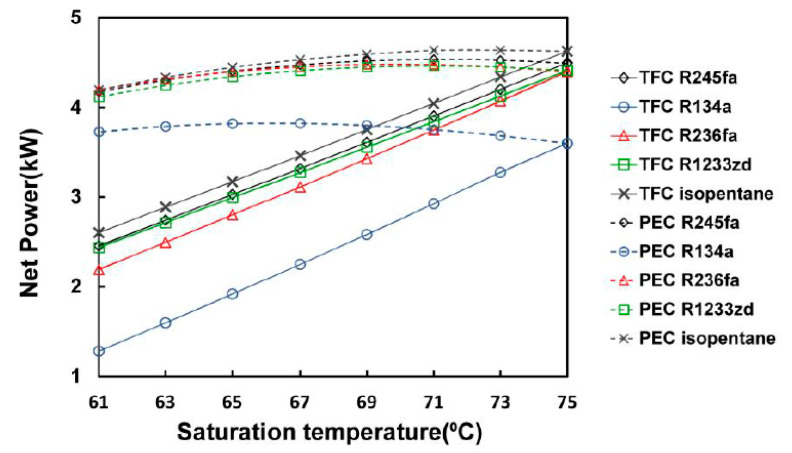
Net power from the TFC and PEC at different saturation temperatures with different working fluids.

After the model was set and the system operation conditions were optimized, the power generated, thermal efficiency, and volume ratios of the five types of working fluids—R245fa, R134a, R236fa, R1233zd, and isopentane—were calculated. Figure 8 presents the effect of the saturation temperature on net power generation, which indicates that the amount of power generated from the TFC was positively correlated with the saturation temperature. As the saturation temperature rose to 75 °C, a greater amount of power generated could be obtained using each working fluid, with isopentane generating the greatest power (4.63 kW), whereas the net power generation of R134a was significantly lower (3.6 kW) because it was limited by a greater operating pressure and the pump consumption. When the saturation temperature decreased by more than 10 °C, the net power generation of each fluid significantly decreased by more than 30%.

In particular, the descent rate of R134a was 46.7%. When the saturation temperature was lower than 63 °C, the descent rate exceeded 50% and resulted in only 1.6 kW. The rise of the saturation temperature of the PEC system presented an optimal value. When the rise was greater than this value, the net power generation of this system dropped. The simulation results indicated that the amount of power generated first increased with the rising saturation temperature and yielded a maximum value at approximately 71 °C (except for R134a with its maximum value occurring at approximately 65 °C). The greatest energy was yielded by isopentane (4.64 kW). In the PEC system, when the saturation temperature dropped to 65 °C, isopentane was the working fluid in which amount of power generated decreased the most; however, the difference was only 3.9%, and the energy generated was sustained at 4.45 kW. The least energy was generated by R134a but still amounted to 3.82 kW. This result suggested that when the saturation temperature decreased, the amount of power generated from the PEC was greater than that of the TFC.

**Figure 9:**
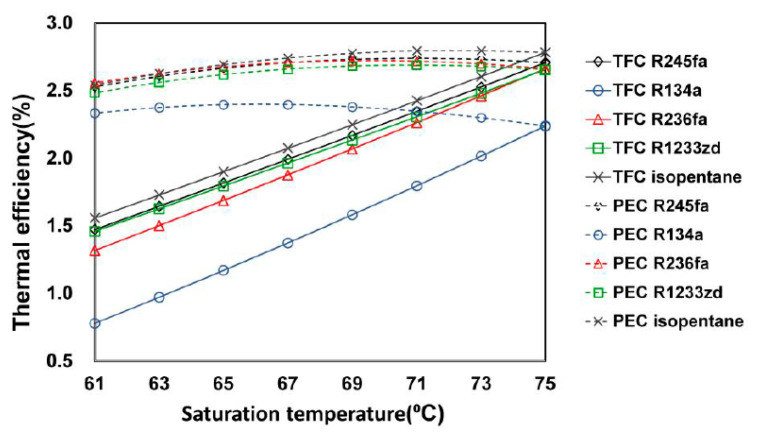
Thermal efficiency for the TFC and PEC at different saturation temperatures with different working fluids.

Figure 9 presents the effect of saturation temperature on thermal efficiency. The variation trend was similar to that of net power generation. When the saturation temperature of the system was 61 °C, the thermal efficiency of each of the five working fluids—R245fa, R134a, R236fa, R1233zd, and isopentane—was 1.47%, 0.78%, 1.32%, 1.46%, and 1.56% (for the TFC) and 2.53%, 2.34%, 2.56%, 2.48%, and 2.54% (for the PEC), respectively. Similar to the net power generation results, after lowering the saturation temperature, the thermal efficiency of each fluid in the PEC system remained considerably higher than that of the TFC system.

**Figure 10:**
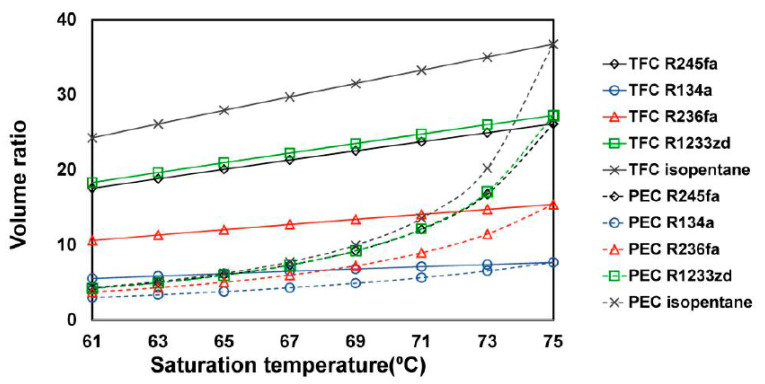
Volume ratio for the TFC and PEC at different saturation temperatures with different working fluids.

Figure 10 presents the effect of the saturation temperature on the volume ratio of the system. The volume ratios of both systems decreased as their saturation temperatures dropped. In the TFC system, when the saturation temperature decreased to 10 °C, only R134a did not have an excessively high volume ratio, whereas the second lowest volume ratio, from R236fa, remained higher than 10. Furthermore, the volume rates of other fluids were more than 15, which indicated that lowering the saturation temperature in the TFC system could not effectively resolve an excessively high volume ratio of the expander. As for the PEC system, lowering the saturation temperature caused the partial evaporation of the working fluid in the heat exchanger, which led to the volume ratio decreasing greatly. When the saturation temperature dropped to 63 °C, the volume ratio of each fluid decreased to approximately 5.

## 5. Conclusions

Among low-grade waste heat recovery techniques, heat energy could not be effectively recovered through the ORC because of its susceptibility to the pinch point, which led to growing attention toward TFC and PEC research to examine the problem of a high volume ratio during the expansion of the TFC system. The characteristics of the TFC and PEC with a low-grade heat source at 80 °C were researched in this study and relationships among net electricity generation, thermal efficiency, and the volume ratio of the TFC and those of the PEC were compared; the findings are as follows:

(1) Lowering the saturation temperature significantly reduced the net power generation and the thermal efficiency of the TFC system; however, the volume ratio could not be reduced effectively. For the PEC system, net power generation and thermal efficiency only decreased slightly, and the volume ratio decreased significantly.
(2) When the saturation temperature of 75 °C dropped by 10 °C, the amount of power generated and the thermal efficiency of the TFC system decreased by more than 20%, and its volume ratio decreased by approximately 20%. The amount of power generated and the PEC system’s thermal efficiency decreased only by 3% at most; however, the volume ratio of the expander decreased by more than 50%.
(3) In practice, if a volume ratio of less than 10 was used to limit the design of the TFC system, only the high-pressure fluid R134a with a lower volume ratio could meet this requirement. Its optimal amount of power generated was 3.6 kW, and its thermal efficiency was 2.24%.
(4) For the PEC system, all working fluids in this study could meet the volume ratio limit simply by lowering the saturation temperature. The most significant amount was yielded by isopentane (4.59 kW), and its thermal efficiency was 2.77%. The performance was significantly better than that of the TFC.
(5) The PEC greatly reduced the volume ratio and reduced the amount of power generated only slightly. This method can be employed to overcome an excessive volume ratio that hinders the turbine’s materialization when developing low-grade heat recovery technology.

## Figures and Tables

**Table 1:** The volume ratio of TFC of different researches.

Authors	Smith [10]		Trædal [14]			Fischer [15]	
Working fluid	water	R113	R245fa	R134a	isopentane	water	isopentane
Heat source	100	100	100	100	100	150	150
Heat sink	20	20	20	20	20	15	15
Volume ratio	~3000	112	26	11	32	2719	76.4

**Table 2:** Input data for the analysis presented in Figure 5 [23].

Pump isentropic efficiency	40%
Inlet water temperature at condenser	15 °C
Expander isentropic efficiency	75%
Heat source	90 °C, 1 kg/s

**Table 3:** Input data for the TFC and PEC.

	Parameter	Value
Heat source	Fluid	Water
	Mass flow	1 kg/s
	Inlet temperature	80 °C
	Pump efficiency	0.70
Heat sink	Fluid	Water
	Inlet temperature	30 °C
Cycle	Type	TFC, ORC
	Working Fluid	R245fa, R134a, R236fa, R1233zd, isopentane
	Saturation temperature	User input
	Mass flow	TFC: Calculated
		PEC: Best case scenario fromTFC
	Vapour quality	TFC: 0
		PEC: Calculated
Heat exchanger	Pinch temperature	5 °C
	Pressure drop	No pressure drop
Expander	Isentropic efficiency, nozzle	0.865 + 0.00175∙$d_{v}$
	Isentropic efficiency, rotor	0.575 + 0.325∙$x_{4}$
Condenser	Pinch temperature	1 °C
	Pressure drop	No pressure drop

## Data Availability

Not applicable.
